# Oral Therapy Using a Combination of Nanotized Antimalarials and Immunomodulatory Molecules Reduces Inflammation and Prevents Parasite Induced Pathology in the Brain and Spleen of *P. berghei* ANKA Infected C57BL/6 Mice

**DOI:** 10.3389/fimmu.2021.819469

**Published:** 2022-01-13

**Authors:** Sitabja Mukherjee, Gopesh Ray, Bhaskar Saha, Santosh K. Kar

**Affiliations:** ^1^ School of Biotechnology, KIIT deemed to be University, Bhubaneswar, India; ^2^ Nano Herb Research Laboratory, Kalinga Institute of Industrial Technology (KIIT) Technology Business Incubator, KIIT deemed to be University, Bhubaneswar, India; ^3^ National Centre for Cell Science, Ganeshkhind, Pune, India

**Keywords:** experimental cerebral malaria (ECM), *P. berghei* ANKA, inflammation control, recrudescence, combination therapy

## Abstract

In malaria, anti-parasite immune response of the host may lead to dysregulated inflammation causing severe neuropathology arising from extensive damage to the Blood Brain Barrier (BBB). Use of anti-malarial drugs alone can control parasitemia and reduce inflammation but it cannot reduce pathology if chronic inflammation has already set in. In the present study, we have tested the efficacy of a new oral artemsinin based combination therapy (ACT) regimen using a combination of anti-malarial compounds like nanoartemisinin and nanoallylated-chalcone9 [{1-(4-Chlorophenyl)-3-[3-methoxy-4-(prop-2-en-1-yloxy) phenyl]-prop-2-en-1-one}]given together with anti-inflammatory-cum- anti-malarial compounds like nanoandrographolide and nanocurcumin to C57BL/6 mice infected with *P. berghei* ANKA. Untreated infected mice developed Experimental Cerebral Malaria (ECM) and died between 10 to 12 days after infection from severe BBB damage. We observed that oral treatments with nanoartemisinin or nano allylated chalcone 9 or nanoandrographolide alone, for 4 days after the onset of ECM, delayed the development of severe neurolopathology but could not prevent it. Nanocurcumin treatment for 4 days on the other hand, prevented damage to the BBB but the mice died because of hyperparasitemia. A single time oral administration of our ACT controlled blood parasitemia and prevented damage to the BBB, but recrudescence occurred due to persistence of parasites in the spleen. However the recrudescent parasites failed to induce ECM and BBB damage, leading to prolonged survival of the animals. A second time treatment at the start of recrudescence led to complete parasite clearance and survival of mice without pathology or parasitemia for 90 days. FACS analysis of spleen cells and gene expression profile in brain and spleen as well as quantitation of serum cytokine by ELISA showed that *P. berghei* ANKA infection in C57Bl/6 mice leads to a Th1-skewed immune response that result in severe inflammation and early death from ECM. Oral treatment with our ACT prevented a heightened pro-inflammatory response by modulating the Th1, Th2 and Treg immune responses and prevented ECM and death.

## Introduction

Malaria continues to be a serious public health problem affecting 241 million people in 2020, causing 627,000 deaths, mostly amongst children from sub-Saharan Africa (1). The primary cause of death in malaria is either severe anemia due to loss of functional healthy erythrocytes from uncontrolled parasite growth in the blood, or complications arising out of imbalance of pro-inflammatory and regulatory immune responses which results in a neuropathological condition known as Cerebral Malaria (CM) (2–4).

Malaria is presently controlled by using effective chemotherapeutic agents. Several trials using natural products and nanoparticles has been attempted to identify new molecules which can be effective against the disease (5, 6). But the parasite develops resistance to the drugs that are used and therefore new therapeutic molecules which can be effective in combination therapy has to be identified and used (7). Artemisinin derivatives such as dihydroartemisinin, artesunate, artemether, and arteether are the most recently developed class of anti-malarial drugs that are being used to control malaria today (8). To avoid the development of resistance to artemisinin-based anti-malarials, the World Health Organization (WHO) recommends that they be used in combination with other anti-malarials as artemisinin based combination therapies (ACTs) (9). However, resistance to existing ACTs has already been reported in multiple locations throughout Southeast Asia where dihydroartemisinin–piperaquine was used as the first line anti-malarial drug (10). Given the past experience with other anti-malarials, resistance to existing ACTs too, is likely to spread globally over time (11–14). This is a cause of concern and therefore new anti-malarial drug combinations have to be identified urgently.

Elimination of the parasite by use of suitable anti-parasitic drugs is crucial in the treatment of malaria. Reduction of parasite burden at an early stage reduces chances of developing excessive inflammation, which in turn reduces the disease pathology. But once the inflammation is set in it cannot be controlled by use of anti-malarial drug treatment alone (15). Therefore, taking suitable measures for prevention of excessive inflammation is necessary to achieve therapeutic efficacy and control of pathology (15, 16). The balance between the beneficial and harmful effects of the anti-malarial immune response elicited by the host is determined by the nature of cytokines that are induced (17). The harmful, dysregulated response is mainly of the Th1 type with over-production of pro-inflammatory cytokines such as IFN gamma, TNF alpha, IL 12, IL 6 etc, combined with under-production of anti-inflammatory cytokines such as IL 10 (17). The role of the immune system is therefore critical in determining the outcomes of malaria infection. An unbalanced immune response is less effective at clearing malaria parasites and may cause damage to vital host tissues leading to severe complications and death (17).

Thus, there has been interest in evaluating the effects of molecules that can modulate the production of different cytokines to eliminate the malaria parasite and alleviate disease pathology (17). In the present study, we attempted to develop an oral therapy for malaria by selecting a combination of compounds with distinct anti-malarial and anti-inflammatory functions to alleviate the severe cerebral pathology in a *P. berghei* ANKA infected C57BL/6 mouse model. We selected artemisinin as it is one of the most potent anti-malarial compounds known that targets the ring and trophozoite stages of the parasite (18). However, since monotherapy is not allowed, and resistance to artemisinin and its derivatives have already been reported, we tried to develop and evaluate the efficacy of a new immunomodulator based ACT treatment, involving artemisinin, allylated chalcone9, andrographolide and curcumin, all of which are reported to have anti-malarial activities. We selected allylated chalcone9 [{1-(4-Chlorophenyl)-3-[3-methoxy-4-(prop-2-en-1-yloxy) phenyl]-prop-2-en-1-one}] as it has not been tested in animal models and is reported to affect different stages of *Plasmodium falciparum* parasite *in vitro*, and can synergize with artemisinin (19). It is also fast acting and can be synthesized easily making it cost effective (19). Andrographolide was selected as it is a natural immunomodulatory compound with anti-malarial activity and is slower acting and therefore can complement the activity of artemisinin (20). Since control of inflammation is essential to reduce pathology associated with malaria infection we selected curcumin as it is a well known natural anti-inflammatory compound which is reported to have synergistic activity with both andrographolide and artemisinin derivatives, and can kill the malaria parasites by inhibition of hemozoin synthesis and disruption of the parasite microtubule assembly (20–22). It has been shown to prevent (Blood Brain Barrier) BBB damage in Experimental Cerebral Malaria (ECM) models in mice (23, 24). We hypothesized that taken together; the immunodulatory component of the oral combination therapy would help to reduce heightened inflammation and prevent induction of CM pathogenesis, while the antimalarial component would be able to reduce the parasite burden efficiently.

## Materials and Methods

### Preparation and Characterization of Artemisinin, Allylated Chalcone9, Andrographolide and Curcumin Nanoparticles

Nanocurcumin (CUR) was prepared following published procedure (25). Briefly, 4 g of curcumin (Sigma, USA) was dissolved in 1L of distilled ethanol at room temperature and filtered to obtain a clear solution. The solution was stirred in a high-speed homogenizer (T 25 digital ULTRA-TURAX, IKA, USA) at 15,160 × g – 23,688 × g, and a required volume of Milli Q water (Merck, India) was added to it slowly over a period of 30 min until the ethanol concentration became 40% (V/V). Gradual addition of water during this stage resulted in the solution mixture becoming more polar, causing a decrease in curcumin solubility and reaching supersaturated conditions at which crystal nucleation leads to curcumin nanoparticle formation. The entire suspension was then homogenized over ice in a high-pressure homogenizer (Avestin C5 High Pressure Homogeniser BPS, UK) at 30,000 PSI for 20 cycles. The aqueous suspension was then made to 0.1% polysorbate 80 (Sigma, USA) and homogenized at 15,160 × g – 23,688 × g (T 25 digital ULTRA-TURRAX, IKA, USA) again for 1 h and filtered. The filtered slurry was dried at 60°C in an oven to get CUR powder.

For preparation of nanoartemisinin (ART) 10 g of artemisinin (Sigma, USA) was first dissolved in 1L of distilled ethanol at room temperature and filtered to obtain a clear solution. The solution was stirred in a high-speed homogenizer (T 25 digital ULTRA-TURAX, IKA, USA) at 15,160 × g – 23,688 × g and a required volume of Milli Q water (Merck, India) was added to it slowly over a period of 30 min until the ethanol concentration became 30% (V/V). The entire suspension was then homogenized over ice in a high-pressure homogenizer (Avestin C5 High Pressure Homogeniser BPS, UK) at 30,000 PSI for 20 cycles. The aqueous suspension was then made to 0.1% polysorbate 80 (Sigma, USA) and homogenized at 15,160 ×g – 23,688 × g (T 25 digital ULTRA-TURRAX, IKA, USA) again for 1 h and filtered. The filtered slurry was dried at 60°C in an oven to get ART powder.

Allylated chalcone 9 was synthesized as previously described (19). Briefly, to a 250 mL round bottom flask containing vanillin (Sigma, USA) (1.9 mmol) in dry acetone (20 mL), allyl bromide (Sigma, USA) (2.0 mmol), and anhydrous K2CO3 [Sigma, (3.8 mmol)] were added. The mixture was refluxed for 6 h. After consumption of aldehyde (monitored by TLC), the mixture was filtered to remove K2CO3. The filtrate was vacuum evaporated and washed with hexane to remove excess of allyl bromide. The crude product was purified by silica gel column chromatography using hexane-ethyl acetate (9:1) to provide 4*-*allyloxy-3-methoxybenzaldehyde. To a solution of 4*-*allyloxy-3-methoxybenzaldehyde (3 mmol) and p-Chloroacetophenone (Sigma, USA) (3 mmol) in methanol (20 mL), 10% aqueous NaOH (4 mmol) was added. The reaction mixture was stirred till completion of starting material (monitored by TLC). The reaction mixture was vacuum evaporated to remove the organic solvent and poured in cold water. The obtained precipitates were washed with dilute HCl, excess of water, methanol, dried in air and finally recrystallized with methanol to obtain pure allylated chalcone 9 (19). After synthesis of the compound, allylated chalcone 9 (4 g) was dissolved in 1L of distilled ethanol at room temperature and filtered to obtain a clear solution. The remaining procedure for synthesis of nano allylated chalcone 9 (AC9), was the same as described for that of CUR preparation.

Andrographolide was procured from Sigma, USA. For preparation of nanoandrographolide (AND), andrographolide (1 g) was dissolved in 1L of boiling distilled ethanol and then filtered to obtain a clear solution. This solution was then stirred in a high-speed homogenizer (T 25 digital ULTRA-TURAX, IKA, USA) at 15,160 × g – 23,688 × g at 60°C. A required volume of Milli Q water (Merck, India) was added to it slowly over a period of 30 min at room temperature until the ethanol concentration became 50% (V/V) and andrographolide nanoparticles started to precipitate from the solution. The remaining procedure for synthesis of nanoandrographolide, was the same as described for that of CUR, ART and AC9 preparation.

The size and morphology of the ART, AC9, AND and CUR was examined using TEM (JEM 2100F, JEOL, USA). One drop of the respective nanoparticles was placed on a carbon coated 300 mesh copper grid and allowed to be air dried. The samples were stained with 1% uranyl acetate added immediately to the surface of the carbon coated grid (25). The particle size distribution was analyzed using dynamic light scattering (Zetasizer Pro, Malvern Panalytical, USA). The intensity of scattered light was detected at 90 degree to an incident beam. The nanoparticles were dispersed in aqueous buffer and measurements were taken after passing the samples through a microfilter having an average pore size of 0.2 mm. The data analysis was performed in automatic mode. Measured size was presented as the average value of 10 runs, with triplicate measurements within each run.

### ECM Model

*P. berghei* ANKA was revived from cryopreserved samples and maintained in 6-8 weeks old female C57BL/6 mice, weighing 18-20 g (26). Blood was collected from infected donor C57BL/6 mice at parasitemia >1% and 1×10^6^ parasitized RBCs (pRBCs) were passaged rapidly into naïve C57BL/6 mice every fourth to fifth day by intraperitoneal (i.p) injection. Briefly, mice were euthanized by carbon dioxide asphyxiation and 500 μl (optimal) blood was collected by cardiac puncture and transferred to 5 ml of RPMI/PS media (RPMI+1% PenStrep) containing 5 μl heparin (5,000 IU/ml) in a 10 ml polypropylene tube. The volume was then made upto 10 ml by adding 5 ml of RPMI/PS media. The cells were then centrifuged at 1,127 x g for 7 minutes at room temperature. The supernatant was then carefully aspirated and the pellet was resuspended in 1 ml RPMI/PS media. An accurate count of pRBC in the suspension was then made using a hemocytometer and an inoculum of 1×10^6^ pRBCs, per 200 μl of RPMI/PS media was intraperitoneally injected into desired number of naïve mice (26). After 3 times passage in donor C57BL/6 mice, 1×10^6^ *P. berghei* ANKA parasites were intraperitoneally injected into experimental C57BL/6 mice (n=12) as described, for the induction of ECM(100%) in infected mice.

### Clinical Score of Disease Severity

Mice were monitored three times daily, and scored on a scale of 0 to 5 (26). The individual symptoms were assigned the following scores- ‘0’ indicated no symptoms; ‘1’ for ruffled fur, ‘1’ for hunching, ‘1’ for wobbly gait, ‘1’ for limb paralysis, ‘1’ for convulsions and ‘1’ for coma. Mice were considered comatose if they were unable to right themselves after being placed on their side. Mice were sacrificed/euthanized by carbon dioxide asphyxiation when the cumulative ECM clinical score reached 4 or above and were assigned a score of 5 at the next time point to denote death. The cumulative score based on the type of symptoms demonstrated was recorded for each mouse daily to generate the average clinical score on each day.

### Single Drug Treatments

The efficacy of single drug treatments were tested in 6-8 weeks old female C57BL/6 mice, weighing 18-20 g. Experimental mice were randomly divided into five different groups (n=12) after infection with *P. berghei* ANKA. The first group was treated orally for 4 days with 200 μL Phosphate Buffer Saline (PBS) per mice from day 6 to day 9 post infection (p.i), and served as the infected-untreated control group. The second, third, fourth, and fifth groups were treated with 50 mg/kg body weight ART, 50 mg/kg body weight AC9, 50 mg/kg body weight AND and 50 mg/kg body weight CUR per mice respectively, orally in 200 μL PBS, from day 6 to day 9 p.i. Apart from these four different groups, a group of healthy uninfected mice were kept as a separate control group (uninfected).

### Combination Treatment

For determining the efficacy of treatment using combination of all the drugs, 6-8 weeks old female C57BL/6 mice, were randomly divided into two groups and infected with 1×10^6^ *P. berghei* ANKA parasites. The infected-untreated group (n=12) was treated with 200 μl PBS from day 6 to day 9 p.i. The infected-combination treated group (n=12) was treated with a combination of 50 mg/kg body weight ART + 50 mg/kg body weight AC9 + 50 mg/kg body weight AND + 50 mg/kg body weight CUR per mice, orally in 200 μl PBS for 4 days from day 6 to day 9 (p.i).

### Effect of Combination Treatmenton Recrudescence

The effect of the combination treatment on parasite recrudescence was tested by infecting mice with *P. berghei* ANKA and treatment with the chosen combination therapy first for 4 days and monitoring for reappearance of parasites followed by a second time treatment again at the time of recrudescence. For this, 6-8 week old female C57BL/6 mice (n=30), were infected with 1×10^6^ *P. berghei* ANKA parasites and then treated with a combination of 50 mg/kg body weight ART + 50 mg/kg body weight AC9 + 50 mg/kg body weight AND + 50 mg/kg body weight CUR per mice, orally in 200 μl PBS first from day 6 to day 9 p.i and again for 4 days at the start of parasite recrudescence.

### Determination of Parasitemia and Survival

Blood parasitemia was determined from thin smears prepared from tail blood followed by fixation in absolute methanol for 1 minute, acridine orange staining and observation in a fluorescent microscope (27). Percentage parasitemia in the blood was calculated according to the formula: [(Number of infected erythrocytes)/(Total number of erythrocytes) × 100%]. The mice were daily monitored for determining the Median Survival Time (MST).

### BBB Permeability

BBB permeability was checked by Evan’s Blue Dye extravasations. The experimental mice were anesthetized and given intravenous injection with 0.2% Evans Blue Dye (Sigma, USA) dissolved in PBS. After 1 hour, the mice were sacrificed and perfused intracardially with PBS. The brains were then surgically removed from these experimental mice, weighed, and the dye was extracted out from the brain tissue samples by keeping the tissues in 100% formamide (Sigma, USA) for 48 h at 37°C. The extracted dye was then quantitated by measuring the absorbance at 620 nm. The concentration of dye per gm of brain tissue was calculated from a standard curve with known concentration of Evan’s Blue in formamide for determining the quantity of Evan’s Blue Dye percolation in the brain tissues in order to assess the damage to the BBB.

### Histological Analysis of Brain and Spleen Tissue

Brain and spleen tissue was collected from uninfected, infected-untreated, and combination treated groups and fixed in 10% buffered formalin (Sigma, USA) solution. The organ specimens were subjected to a tissue processor using an automated tissue processor (Leica, Germany) to remove the water from the tissues. The specimens were embedded into melted paraffin wax using a histoembedder (Leica, Germany), sectioned into a 4.0μm thick slice with a microtome (Leica, Germany) and stained with hematoxylin and eosin (H&E) using an autostainer. The morphological changes within the tissues of the different groups were observed under a light microscope (under 40×magnification).

### FACS Analysis

Spleen tissue was collected from the uninfected, infected-untreated and infected-combination treated mice. Splenocytes sans erythrocytes were isolated and blocked with 30% FBS in PBS for 30 min at 4°C. The cells were washed with 2 ml PBS, counted and distributed 1x10^6^ cells/tube. The samples were stained with a cocktail of anti-mouse -CD3-FITC (Biogene India, CAT no. 100203) and anti-mouse -CD4-PB antibodies (Biogene India, CAT no. 100427). For Treg set, the samples were additionally stained with anti-mouse CD25-APC-Cy7 antibody (Biogene India, Cat no. 102025). Following surface staining, the samples were permeabilized and intracellular staining for the different sets was done using anti mouse Tbet-APC conjugated antibodies (Biogene India, Cat no. 644813) or anti -mouse GATA3-APC conjugated antibodies (Biogene India, Cat no. 653805) or anti-mouse Foxp3-Alexa Fluor^®^ 647 conjugated antibodies (Biogene India, Cat no. 320013), respectively. Following intracellular staining, the cells were fixed with 1% paraformaldehyde (Sigma, USA) and stored in dark at 4°C till FACS analysis.

### Analysis of Gene Expression Profile in the Brain and Spleen

Brain and spleen tissue were harvested from experimental mice and RNA was extracted from brain and spleen samples using TRIzol (TRI reagent, Invitrogen) and 1μg of RNA was taken for reverse transcription reaction using Invitrogen SuperScript II Reverse Transcriptase kit according to manufacturer’s protocol. The prepared cDNA was diluted 1:20 times and used for quantitative polymerase chain reaction (qPCR) in Appplied Biosystems Step One Plus Real Time PCR System under the following conditions: 95°C for 2 min, 50 cycles of 95°C for 1 min, 60°C for 30 s, 72°C for 35 s. Glyceraldehyde 3-phosphate dehydrogenase (GAPDH) was kept as the endogenous control and relative fold expression was calculated by comparative threshold (2^-ΔΔCT^) method (27).

### Serum Cytokine Analysis by ELISA

Blood was collected from mice by retro-orbital bleeding and serum was prepared by allowing clot formation to occur over a period of 15 minutes at room temperature followed by removal of clot by centrifugation at 1000 X g for 10 mins at 4°C and collecting the supernatant fraction (serum) into fresh autoclaved polypropylene tubes while keeping on ice. Cytokine levels in the serum were measured by using DuoSet mouse ELISA kits (R&D systems, USA) as per the manufacturer’s protocol. Biotinylated detection antibody and avidin-HRP conjugate were used with (3,3’,5,5’-Tetramethylbenzidine) (TMB) liquid substrate to give a colored product. Reaction was stopped using 2N H _2_SO _4_ and absorbance at 450 nm was measured to detect and quantitate the levels of IFN gamma, TNF alpha, IL 12, IL 6, IL 4 and IL 10 in the serum.

### Animal Ethics Approval

Mice were used according to the approval (No. IEAC/2017/B-287) by the Institute’s Animal Care and Use Committee (IACUC), National Centre for Cell Science, Pune and by the Committee for the Purpose of Control and Supervision of Experimental Animals (CPCSEA) India.

### Statistical Analysis

Statistical analysis was performed by using GraphPad Prism software (GraphPad Prism v 5.0). Statistical difference between all the groups was determined by One-way Anova by Bonferroni’s multiple comparison test. The MST (95% CI) was determined using Log-rank (Mantel-Cox) Test in GraphPad Prism software (GraphPad Prism v 5.0). P<0.05 was considered significant.

## Results

### Characterization of ART, AC9, AND & CUR

**Figure 1:**
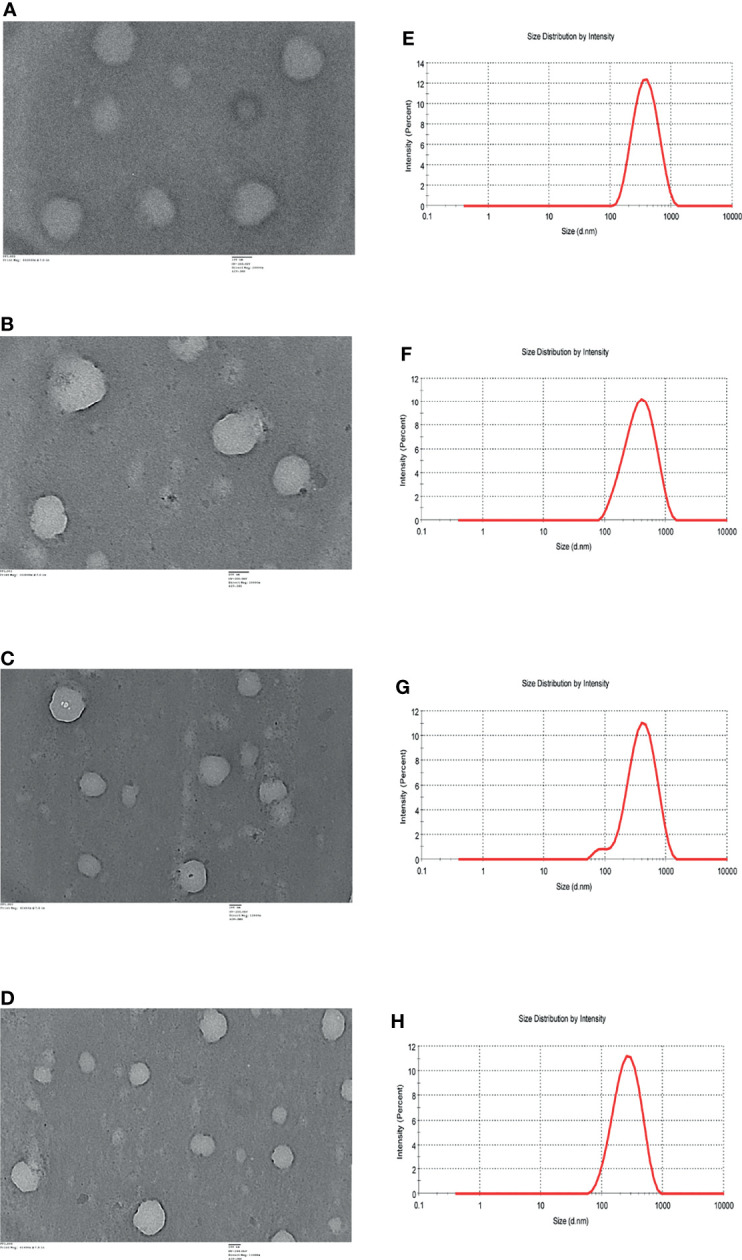
TEM images of **(A)** nanoartemisinin (ART) **(B)** nano-allylated chalcone9 (AC9) **(C)** nanoandrographolide (AND) **(D)** nanocurcumin (CUR). DLS profiles of **(E)** ART **(F)** AC9 **(G)** AND **(H)** CUR.

TEM analysis of ART (**Figure 1A**), AC9 (**Figure 1B**), AND (**Figure 1C**), and CUR (**Figure 1D**) demonstrated that they are spherical nanoparticles having a size of 100 nm or above. The average size (hydrodynamic radius) of ART (**Figure 1E**), AC9 (**Figure 1F**), AND (**Figure 1G**), and CUR (**Figure 1H**) was determined to be 327 ± 180.7 nm, 331 ± 221.9 nm, 335.2 ± 215.8 nm and 230.5 ± 137.7 nm respectively; with a polydispersity index (PdI) of 0.197, 0.219, 0.227 and 0.180 respectively by dynamic light scattering analysis.

### Infection With *P. berghei* ANKA Causes Induction of ECM

**Figure 2:**
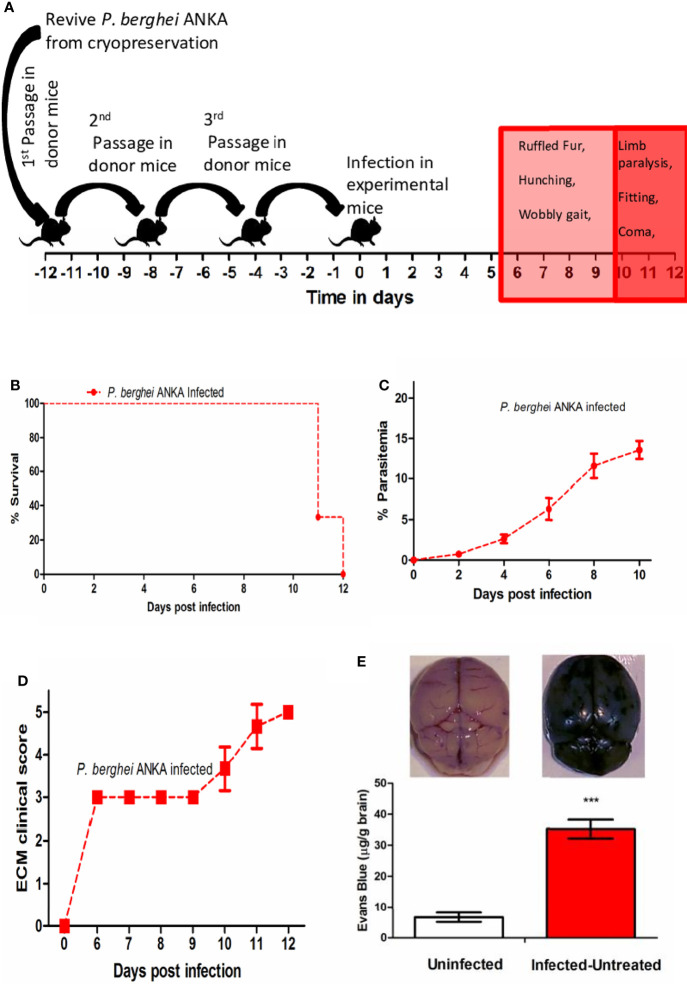
**(A)** Schematic for establishment of ECM in C57BL/6 mice. **(B)** Survival curve **(C)** Trend in parasitemia and **(D)** ECM clinical score of *P. berghei* ANKA infected C57BL/6 mice. Data is representative of 3 independent experiments **(E)** Quantitation of Evan’s Blue Dye extravasation in the brain of Uninfected vs Infected-untreated mice on day 10 p.i. Data is expressed as mean ± SD from 3 mice per group. [*P<0.05, **P<0.01, ***P<0.001, n.s., not significant].

In preliminary studies we observed that cryopreserved *P. berghei* ANKA parasites when injected directly to naïve mice led to ECM induction in 66.6% of the infected mice that died between 10 to 12 days p.i (**Table 1**). The minimum number of passages required for ECM induction in 100% of infected mice was determined from preliminary studies to be 3 (**Table 1**). After 3 times passage in donor mice at >1% parasitemia, injection of 1X10^6^ parasitized RBCs (pRBCs) into experimental mice led to induction of ECM symptoms in the infected mice from day 6 p.i, with the symptoms becoming severe between days 10 to 12 p.i (**Figure 2A**). The MST for infected mice was observed to be 11 days p.i (**Figure 2B**). A steady rise in parasitemia was observed starting from day 2 p.i, reaching an average parasitemia of 6.33% ± 1.35 on day 6 p.i and 13.567% ± 1.12 on day10 p.i (**Figure 2C**). The infected mice started demonstrating ECM symptoms starting from day 6 p.i with a clinical score of 3 which reached an average of 3.667 ± 0.516 by day 10 p.i (**Figure 2D**). The infected mice started demonstrating severe neurological symptoms like limb paralysis, convulsions and coma from day 10 p.i when the ECM clinical score reached >3 (**Figure 2D**). Quantification of Evan’s Blue dye in the brain, demonstrated that there was a significant increase in the amount of the dye that percolated into the brain of infected mice in comparison to uninfected control mice, indicating extensive damage to the BBB in the infected mice (**Figure 2E**).

**Table 1:** *P. berghei* ANKA was revived from cryopreserved samples and maintained in 6-8 weeks old female C57BL/6 mice (n=12).

Number of passages	Number of mice showing ECM symptoms	% ECM	Time of death from ECM (days p.i)
1	8/12	66.66%	10-12
2	10/12	83.33%	10-12
3	12/12	100%	10-12

Blood was collected from infected C57BL/6 mice at >1% parasitemia and 1×10^6^ parasitized RBCs (pRBCs) were passaged rapidly into naïve C57BL/6 mice every fourth to fifth day by i.p injection. The number of mice that developed neurological symptoms and succumbed from ECM was monitored. The minimum number of passages that were required for induction of ECM into 100% of the mice was recorded.

### ART, AC9 & AND Treatment Delays ECM While CUR Treatment Prevents BBB Damage

**Figure 3:**
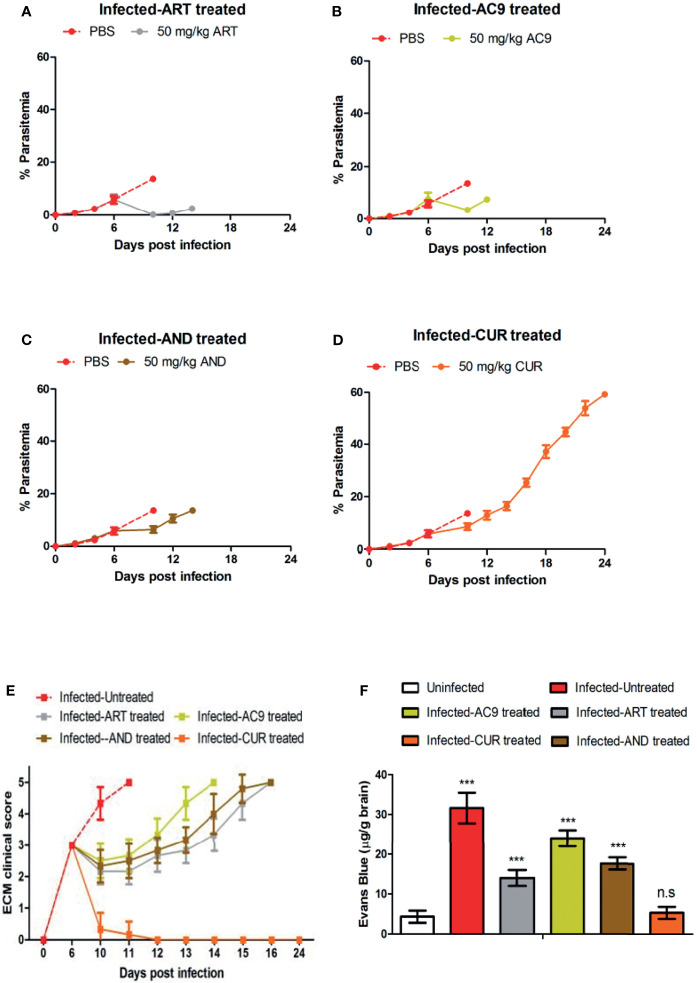
Efficacy of single drug treatments with ART, AC9, AND or CUR in *P. berghei* ANKA infected C57BL/6 mice was evaluated. Infected-untreated mice were treated with only PBS. Parasitemia of **(A)** infected-untreated vs infected-ART treated group, **(B)** infected-untreated vs infected-AC9 treated group, **(C)** infected-untreated vs infected-AND treated group and **(D)** infected-untreated vs infected-CUR treated group was recorded. **(E)** The ECM clinical score for infected-untreated, infected-ART treated, infected-AC9 treated, infected-AND treated, infected-CUR treated groups were monitored and **(F)** damage to the BBB was assessed by quantitation of Evan’s Blue Dye in the brain when the ECM clinical score for infected-untreated group reached 4. Parasitemia, survival curve and ECM clinical score data is representative of 3 independent experiments and data from 6 mice per group has been represented. Evan’s Blue Dye quantitation data is expressed as mean ± SD from 3 mice per group. [*P<0.05, **P<0.01, ***P<0.001, n.s., not significant].

The treatment of infected mice with individual compounds for 4 days was started from day 6 p.i after the onset of ECM symptoms when the clinical score reached 3. Only in the infected-ART treated group the parasitemia was reduced to undetectable levels on day 10 p.i, in comparison to 13.63% ± 0.66 in the infected-untreated mice; accompanied with parasite recrudescence by day 12 p.i (**Figure 3A**). Treatments of infected mice only with AC9 or AND or CUR for 4 days reduced the parasitemia in the blood to 3.38% ± 0.82, 6.40% ± 1.26 and 8.57% ± 1.23 respectively by day 10 p.i (**Figures 3B–D**). When ECM clinical score was recorded, we observed that treatment with ART or AC9 or AND alone could only delay the development and severity of ECM symptoms in comparison to infected-untreated group but could not prevent it, whereas treatment with CUR alone completely reversed and prevented it (**Figure 3E**). Quantification of Evan’s Blue dye percolation to the brain on day 10 p.i, showed that there was a significant increase in the BBB permeability in the groups infected- untreated or infected and treated with ART only or AC9 only or AND only in comparison to uninfected group, whereas the change of BBB permeability in the infected-CUR treated group was unaltered (**Figure 3F**). The MST for infected-untreated, infected-ART treated, infected-AC9 treated, infected-AND treated and infected-CUR treated groups were observed to 11, 16, 14, 15 and 24 days p.i respectively (**Table 2**).

**Table 2:** Experimental mice were randomly divided into five different groups (n=12) after infection with 1X10^6^ *P. berghei* ANKA.

Groups	Treatment	MST (days p.i)
1	Infected-Untreated	11
2	Infected-ART treated	16
3	Infected-AC9 treated	14
4	Infected-AND treated	15
5	Infected-CUR treated	24

The first group was treated orally for 4 days with 200 μL PBS per mice from day 6 to day 9 p.i. The second, third, fourth and fifth groups were treated with 50 mg/kg body weight ART per mice, 50 mg/kg body weight AC9, 50 mg/kg body weight AND and 50 mg/kg body weight CUR respectively, orally in 200 μL PBS, from day 6 to day 9 p.i. The survival period for all the groups was recorded and MST from 6 mice per group was tabulated.

### Combination Treatment Delays Recrudescence & Prevents Disease Severity

**Figure 4:**
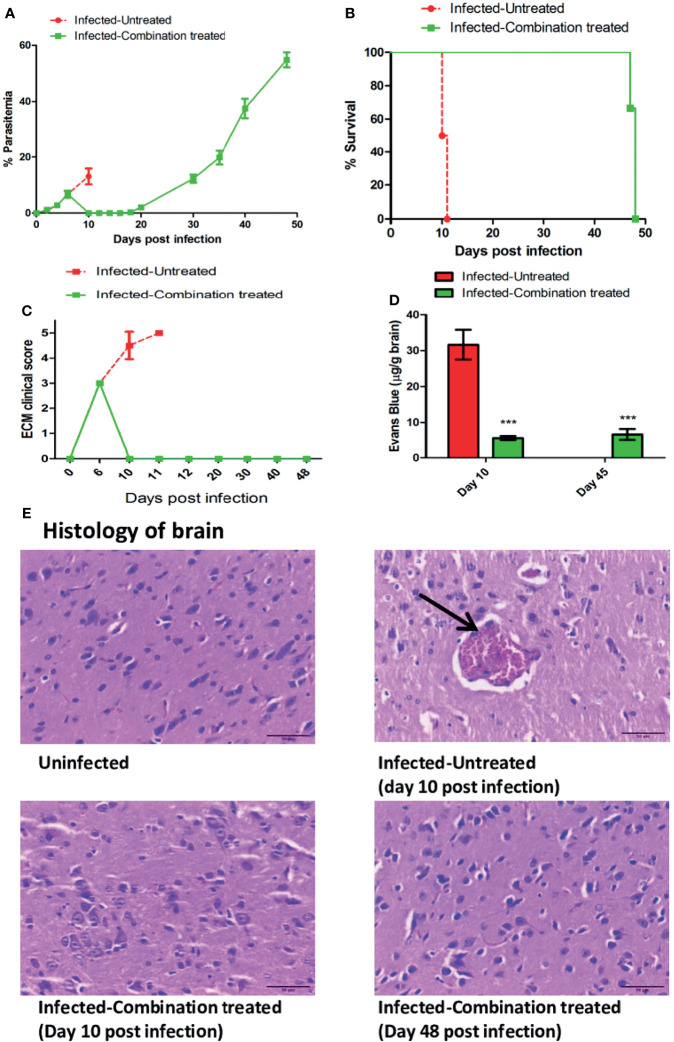
Efficacy of treatment with a combination of ART+AC9+AND+CUR in *P. berghei* ANKA infected C57BL/6 was evaluated. Infected-untreated mice were treated with only PBS. **(A)** Trend in parasitemia, **(B)** Survival curve, **(C)** ECM clinical score and **(D)** Quantitation of Evan’s Blue dye extravasation in the brain of infected-untreated vs infected-combination treated group was evaluated. Parasitemia, survival curve and ECM clinical score data is representative of 3 independent experiments and data from 6 mice per group has been represented. Evan’s Blue Dye quantitation data is expressed as mean ± SD from 3 mice per group. [*P<0.05, **P<0.01, ***P<0.001, n.s., not significant]. **(E)** Histology of cortical sections of brain from uninfected, infected-untreated and infected-combination treated groups. Black arrow indicates the sequestration of infected erythrocytes in the brain microvasculature. Scale bar: 30μm.

Since treatment with the individual compounds was successful in delaying death, we tested the efficacy of combining all the compounds together and administering them orally for 4 days. We observed that blood parasitemia was suppressed and undetectable in the infected-combination treated group from day 10 to 16 p.i. Parasite recrudescence was observed on day 18 p.i and reached 54.88% ± 2.66 by day 48 p.i (**Figure 4A**). The MST of the infected-combination treated group was 48 days (**Figure 4B**). Interestingly, the ECM clinical score remained undetectable even in presence of high parasitemia in the blood (**Figure 4C**). BBB damage for was assessed first on day 10 p.i when the average ECM clinical score for infected-untreated group was >4 and again on day 45 p.i when hyperparasitemia (>40%) in the blood of the infected-combination treated group was observed. Evan’s blue dye quantitation in the brain demonstrated that percolation of the dye in the brain of infected-combination treated group was significantly lower than infected-untreated group on both day10 p.i, as well as on day 45 p.i (**Figure 4D**). Histology of the brain tissue was similarly performed first on day 10 p.i, and again on day 48 p.i at the time of death in the infected-combination treated group. Histological analysis of brain sections (28) showed that the brain of infected-combination treated group were devoid of parasite sequestration on both day 10 p.i and also on day 48 p.i as compared to infected-untreated group which showed extensive sequestration of pRBCs (**Figure 4E**, **Table 3**).

**Table 3:** Experimental mice were randomly divided into 2 different groups (n=12) after infection with 1X10^6^ *P. berghei* ANKA.

Groups	Dilation & congestion of capillaries	Hemorrhages	Durck granuloma	Extravasation of RBCs	Parasitized RBCs	Cerebral oedema
Uninfected	Absent	Absent	Absent	Absent	Absent	Absent
Infected-untreated (day 10 p.i)	3+ to 4+	1+, focal	Absent	1+	2+ to 3+	1+, focal
Infected- combination treated (day 10 p.i)	Absent	Absent	Absent	Absent	Absent	Absent
Infected-combination treated (day 48 p.i)	Absent	Absent	Absent	Absent	Absent	Absent

The first group (infected-untreated) was treated orally for 4 days with 200 μL PBS per mice from day 6 to day 9 p.i. In the second group, treatment with a combination of ART+AC9+AND+CUR was given from day 6 to 9 p.i. The histological findings between the groups in comparison to uninfected (control) group in the whole-brain of mice (3 mice per group) were tabulated. + is used to denote the severity and extent of the parameters at the site according to the following scale:- 1+ means < 25% RBCs are parasitized, 2+ means 25-50% RBCs are parasitized, 3+ means 50-75% RBCs are parasitized, 4+ means > 75% RBCs are parasitized, Absent - 0% RBCs parasitized.

### Combination Treatment Prevents Disease Severity by Reducing a Th1 Skewed Immune Response

**Figure 5:**
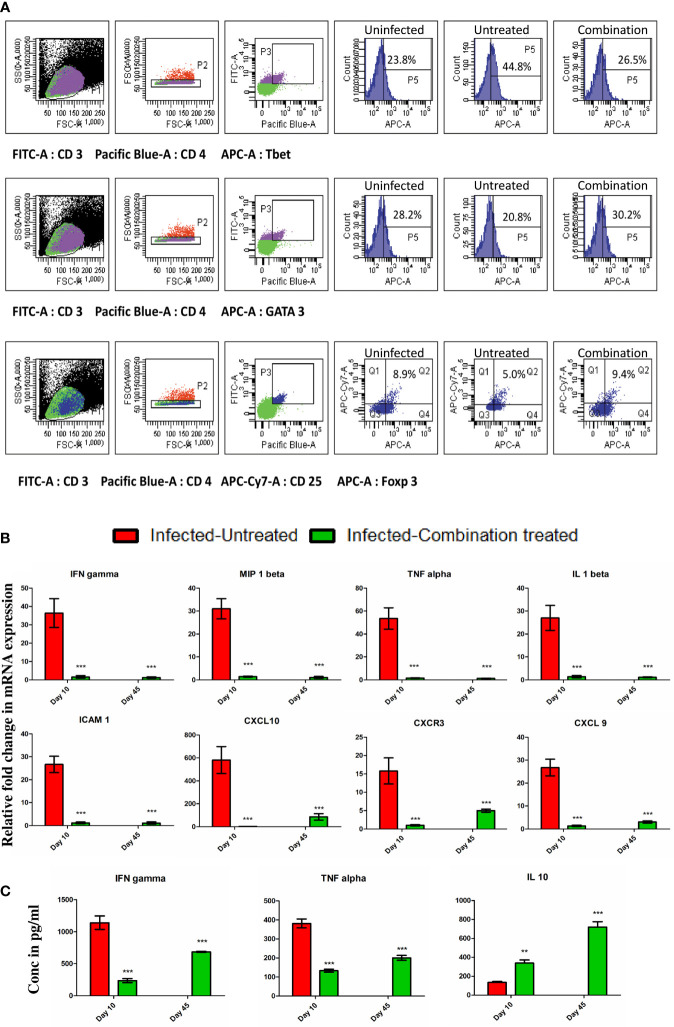
**(A)** Splenocytes were isolated from the spleen of uninfected, infected-untreated, and infected-combination-treated groups on day 10 p.i. Surface staining was performed with anti-mouse CD3-FITC conjugated antibodies, anti-mouse CD4-PB conjugated antibodies and anti-mouse CD25-APC-Cy7 conjugated antibodies. Intracellular staining with anti-mouse Tbet-APC conjugated antibodies, or anti-mouse GATA3-APC conjugated antibodies or anti-mouse Foxp3- Alexa Fluor^®^ 647 conjugated antibodies was performed to determine the percentages of CD3+ CD4+ Tbet+ T cells, CD3+ CD4+ GATA3+ T cells and CD4+CD25+Foxp3+ T cells in spleen. **(B)** Gene expression of IFN gamma, MIP 1beta, TNF alpha, IL 1beta, ICAM 1, CXCL 10, CXCR3 and CXCL 9 in the brain of infected-untreated vs infected-combination treated group on day 10 and 45 p.i. Data is expressed as mean ± S.D of relative fold change in mRNA expression from 3 mice per group **(C)** Serum cytokine analysis of IFN gamma, TNF alpha and IL 10 levels of infected-untreated vs infected-combination treated group on day 10 and 45 p.i. Data is expressed as mean ± S.D of serum cytokine concentrations from 3 mice per group. [*P<0.05, **P<0.01, ***P<0.001, n.s., not significant].

We observed that in the infected-untreated group, the percentage of CD3+CD4+ Tbet+T cells in the spleen increased to 44.8% as compared to 23.8% in uninfected group on day 10 p.i (**Figure 5A**). At the same time, the percentage of CD3+CD4+GATA3+ T cells and CD3+CD4+CD25+Foxp3 T cells in the spleen of infected-untreated group decreased to 20.8% and 5.0% respectively in comparison to 28.2% and 8.9% in the uninfected group (**Figure 5A**). By contrast, the percentage of CD3+CD4+Tbet+ T cells, CD3+CD4+GATA3+ T cells, and CD3+CD4+CD25+Foxp3 T cells in the spleen of infected-combination treated group were 26.5%, 30.2% and 9.4% respectively (**Figure 5A**). The expression levels of IFN gamma, MIP 1beta, TNF alpha, IL 1beta, ICAM 1, CXCL 10, CXCR3 and CXCL 9 in the brain of infected-combination treated group on both day 10 and day 45 p.i were observed to be significantly lower than that of infected-untreated group by qPCR analysis (**Figure 5B**). Similarly the levels of pro-inflammatory cytokines IFN gamma and TNF alpha in the serum of infected-combination treated group was found to be significantly lower than that of infected-untreated group whereas the level of anti-inflammatory cytokine IL 10 was significantly higher (**Figure 5C**).

### Two-Time Combination Treatment Prevents Recrudescence

**Figure 6:**
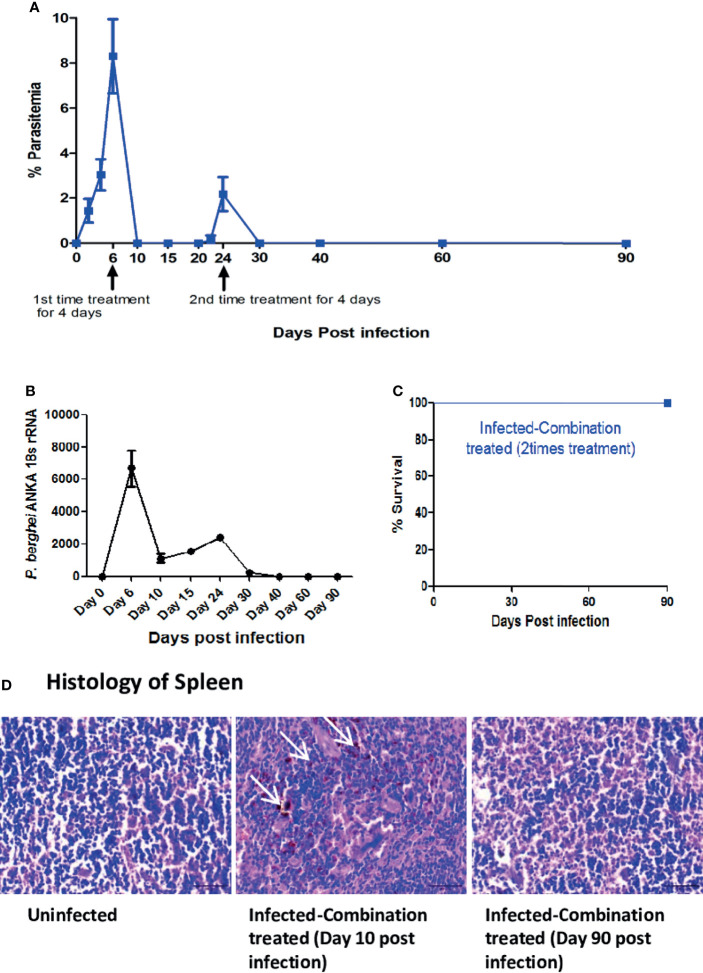
Efficacy of two-time treatment with a combination of ART+AC9+AND+CUR in *P. berghei* ANKA infected C57BL/6 was evaluated. Treatment with a combination of ART+AC9+AND+CUR was first given from day 6 to 9 p.i and again from day 24 to 27 p.i. after detection of recrudescence, followed by monitoring the **(A)** Trend in Parasitemia **(B)** *P. berghei* ANKA 18s rRNA levels in spleen and **(C)** Survival curve. Parasitemia, and survival curve data is representative of 3 independent experiments and data from 6 mice per group has been represented. *P. berghei* ANKA 18s rRNA levels have been quantitated from 3 mice per group and data is representative of 3 independent experiments. **(D)** Histology of spleen sections from uninfected and infected-combination treated group on day 10 and day 90 p.i. White arrows indicate the sequestration of infected erythrocytes in the sinusoids. Data is representative of 3 independent experiments. Scale bar: 30μm.

**Figure 7:**
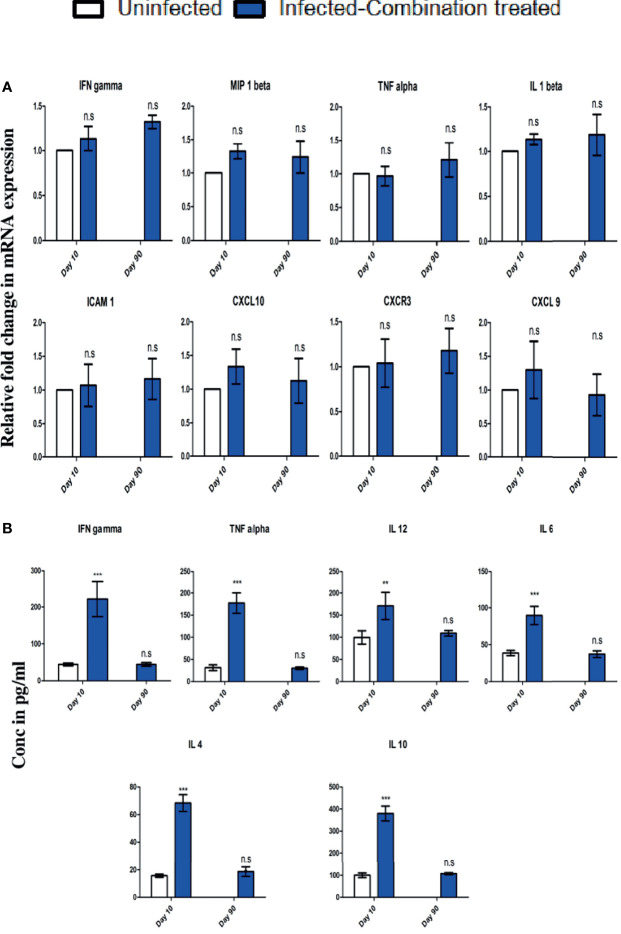
C57BL/6 mice were infected and treated as described in legend for . **(A)** Changes in gene expression of IFN gamma, MIP 1beta, TNF alpha, IL 1beta, ICAM 1, CXCL 10, CXCR3 and CXCL 9 in the brain of infected-combination treated group on day 10 and day 90 p.i in comparison to uninfected group. Data is expressed as mean ± S.D of relative fold change in mRNA expression from 3 mice per group. **(B)** Changes in serum cytokine levels of IFN gamma, TNF alpha, IL 12, IL 6, IL 4 and IL 10 of infected-combination treated group on day 10 and day 90 p.i in comparison to uninfected group. Data is expressed as mean ± S.D of serum cytokine concentrations from 3 mice per group. [*P<0.05, **P<0.01, ***P<0.001, n.s., not significant].

Since recrudescence occurred after single time combination treatment, we decided to administer the treatment a second time for 4 days after recrudescence was observed to occur in all the mice in the group. We observed that second time treatment led to complete suppression of parasites in the blood with no detectable parasitemia till day 90 p.i at which point the observation was terminated (**Figure 6A**). For understanding the cause of parasite recrudescence, we quantitated the *P. berghei* ANKA 18s rRNA levels in the spleen as *P. berghei* ANKA has been previously reported to remain sequestered in the spleen from where it comes back into the peripheral circulation. We observed that single time dosing for 4 days with combination treatment significantly reduced the *P. berghei* ANKA 18s rRNA levels in the spleen on day 10 p.i, but did not lead to complete clearance even though parasite could not be detected in the blood (**Figure 6B**). There was an increase in the *P. berghei* ANKA 18s rRNA levels in the spleen by day 24 p.i corresponding with a blood parasitemia of 2.17% ± 0.75 at the recrudescent phase. After administering a second dose of combination treatment for 4 days from day 24 p.i the *P. berghei* ANKA 18s rRNA levels in the spleen and the parasitemia in the blood was reduced to undetectable levels by day 30 p.i (**Figure 6B**). The treated mice survived till day 90 p.i at which point the observation was terminated (**Figure 6C**). Histological analysis of spleen sections confirmed the presence of parasite infected RBCs sequestered in the spleen of infected-combination treated group on day 10 p.i (**Figure 6D**). By day 90 p.i, the splenic architecture was devoid of parasite infected RBCs. Analysis of expression of IFN gamma, MIP 1beta, TNF alpha, IL 1beta, ICAM 1, CXCL 10, CXCR3 and CXCL 9 by qRTPCR in the brain of infected-combination treated group, showed that there was no significant change on day 10 p.i or day 90 p.i with respect to uninfected control group (**Figure 7A**). The levels of IFN gamma, TNF alpha, IL 12, IL 6, IL 4 and IL 10 in the serum of infected-combination treated group were significantly higher than uninfected group on day 10 p.i but by day 90 p.i the changes in the levels of IFN gamma, TNF alpha, IL 12, IL 6, IL 4 and IL 10 in the serum between the infected-combination treated group and uninfected control were statistically non-significant (**Figure 7B**).

## Discussion

Heightened inflammation induces breach of BBB and entry of parasitized red blood cells into the brain causing severe pathology and neurological damage of the brain which is termed as Cerebral Malaria (29, 30). Using *P. berghei* ANKA infected C57BL/6 mice models it has been established that infection in these mice induces a condition called ECM arising out of neurological damage with motor dysfunction, convulsions and coma (31). *P. berghei* ANKA infection in C57BL/6 mice is the model which has been extensively used to study cerebral malaria. It also manifests many of the symptoms and pathologies observed in most severe malaria syndromes in humans (31). Studies involving animal models has become a necessity because ethical considerations does not allow use of human tissue.The C57BL/6 mice show progressive behavioural, histopathological and immunological changes after infection with *P. berghei* ANKA parasites which results in early death even at low parasitemias. Infected mice that survive the window of severe neurological symptoms between days 6 and 10 p.i. still become moribund around 20 days p.i, displaying evidence of anemia and hyperparasitemia. The *P. berghei* ANKA infection in C57BL/6 mice thus bears striking similarities to the human disease, making it a very useful and suitable model for study of malaria (31). In our study, we also observed the onset of ECM symptoms in infected-untreated mice like fur ruffling, hunching and wobbly gait from day 6 p.i which then progressed to more severe symptoms like convulsions, paralysis and coma by day 10 p.i and ultimately death within 12 days p.i from extensive BBB damage (32). Earlier studies have shown that i.p injections of 30 mg/kg body weight artesunate for 6 days at the onset of neurological symptoms, failed to provide complete protection against ECM in *P. berghei* ANKA infected C57BL/6 mice (33). This is similar to what is observed in human cerebral malaria (HCM) when treatment fails. Similar studies have demonstrated that i.p injection of 5 mg/kg body weight artimisone administered as split doses from day 3 to 6 p.i delayed ECM in mice, reduced parasitemia but did not prevent onset of ECM (34). However when a higher dose of 10 mg/kg body weight of artemisone was given it completely cured the mice from infection and prevented ECM (34). It is clear that eliminating the parasite should be an essential step in treatment of malaria therefore experimental studies and clinical practice so far, have focused on reduction of parasite load by using anti-malarial drugs (17). But in some patients, death occurs despite anti-parasitic treatment (35). One possible cause of death associated with severe neurological manifestations of malaria can be attributed to cerebral inflammation resulting from an imbalance in the production of cytokines and neurotoxic factors that is triggered by the parasite (35). As a drug with combined anti-parasitic and anti-inflammatory effects, curcumin has been previously used in a non-CM model of *P. berghei* infection in Swiss mice and has been demonstrated to decrease parasitemias by 80% and enhance survival when administered orally at a dose of 100 mg/kg body weight (36). In a similar study, oral treatment with curcumin at 50 mg/kg body weight twice a day, for 7 days prevented CM in *P. berghei* ANKA infected C57BL/6 mice and delayed death without affecting parasitemia (17, 24). Oral administration of curcumin bound to PLGA nanoparticles was also observed to not prevent parasitemia but reduced inflammation and prevented cerebral malaria pathology and prolonged survival of mice from 10-12 days to 22-25 days p.i (37). Curcumin was demonstrated to prevent CM in *P. berghei* ANKA infected mice by reducing inflammation and preventing sequestration of CD8+T cells in the brain (37). Combination of i.p injection of arteether and 3 oral doses of curcumin reduced parasite burden and prevented CM to prolong survival, in an associated study (38). A dysregulated and heightened pro-inflammatory response is responsible for development of ECM and severe neuropathological damage in *P. berghei* ANKA infected C57BL/6 mice, because of the breakdown of the BBB. Curcumin is a natural, dietary, non-toxic compound and it exhibits anti-malarial as well as potent anti-inflammatory activity. Previous observations involving use of curcumin have indicated that a combination of immunomodulators with anti-malarial drugs has the potential to improve outcomes in malaria infection (17, 37, 38). Since treatment with antimalarials alone can’t prevent pathology arising from inflammatory damage to vital organs once chronic inflammation has already set in, treatment with an anti-inflammatory compound like curcumin has considerable appeal as an important component of the immunomodulator based ACT envisaged in this study. Curcumin has been classified as a “Generally Recognized As Safe (GRAS) molecule and shown to be effective against different types of infectious diseases and inflammatory conditions (39). Several human clinical trials has shown its suitability as an effective immunomodulator for treatment of malaria (39). In our study, we therefore attempted to develop an oral therapy regimen comprising of a combination of nanoartemisinin, nano allylated chalcone9, and nanoandrographolide together with nanocurcumin which when given orally, can prevent severe cerebral malaria pathology. The doses and route of administration was determined after careful consideration in accordance to published studies (40–44). Since we were interested in protection against ECM, the drugs were administered after the onset of neurological symptoms leading to ECM. We observed that oral treatment of mice with nano forms of artemisinin, allylated chalcone9 and andrographolide individually at 50 mg/kg body weight for 4 days, only delayed the onset of severe neurological symptoms but could not prevent it even though it reduced the blood parasitemia. On the other hand, oral treatment with 50 mg/kg body weight nanocurcumin alone cured all the neurological symptoms and prevented ECM but the animals died later due to hyperparasitemia (>40%) as it failed to control parasites in the blood. This has also been observed by others in previous studies (37, 38). This demonstrated that prevention of neuro-pathological conditions by nanocurcumin treatment, can prolong the survival of mice. Therefore when all the 4 compounds were given in combination for 4 days, it led to prevention of ECM and undetectable blood parasitemia but recrudescence occurred 10 days after the treatment was stopped. Earlier studies have indicated that *P. berghei* ANKA parasites infect early reticulocytes and establish a cryptic asexual cycle in the major sites of haematopoiesis and can persist within early reticulocytes in the spleen even after getting cleared from peripheral blood after treatment with artemisinin (45). Histopathological analysis of the spleen demonstrated that in *P. berghei* ANKA infected mice, there was enlargement of red and white pulp regions accompanied by the loss of the structure of germinal centre in comparison to uninfected mice (**Figure S1**). The red pulp serves as an iron recycling site, and a site for macrophages to remove senescent and pRBCs during infection whereas the white pulp is a site for antigen presentation and activation of adaptive immune responses (46). The white pulp comprises of B and T cell regions and under normal conditions is separated from the red pulp by a marginal zone. The spleen of uninfected control mice presented distinct T-cell and B-cell zones in the white pulp which was surrounded by well-defined marginal zones (**Figure S1**, **Table S1**). By comparison, in the infected-untreated mice, there was considerable proliferation of cells in the white pulp along with enlargement of the white pulp region. This resulted in a loss of distinction between the white pulp and red pulp (**Figure S1**, **Table S1**). There was also accumulation of pRBCs together with heavy deposition of the hemozoin and presence of hemozoin laden monocytes and macrophages in the red pulp sinusoids of infected-untreated mice which contributed to the loss differentiation (**Figure S1**).The cellular expansion of the red pulp and white pulp regions take place as a response to control the infection by clearance of parasite, immune cell activation and regulation of hematopoiesis (46). However, since the spleen acts as a front line organ for tackling the infection, chronic pro-inflammatory conditions lead to a disarray of the splenic architecture (46). As defined regions in the white pulp and red pulp play a crucial role in immune activation and controlling the blood stage infection, disorganization of the architecture can disrupt key interactions between diverse cells. This may lead to potentially deleterious consequences (46). After the single time treatment with the combination therapy, splenomegaly was reduced in the infected-treated mice in comparison to infected-untreated mice but was significantly higher than that of uninfected mice (**Table S2**). The loss of differentiation of the splenic architecture was far less pronounced as compared to infected-untreated mice but there was still marked enlargement in the red pulp and a more prominent white pulp, together with deposition of hemozoin, pigment laden macrophages and presence of infected RBCs (**Figure S1**, **Table S1**). Studies have suggested that after artemisinin treatment, a small percentage of parasites enter a dormant stage and become unresponsive to the treatment (47). These parasites can resume normal development and result in detectable parasitemia when treatment is discontinued (47–49). Therefore, we used the strategy of administering a first time treatment to prevent ECM by reducing blood parasitemia and excessive inflammation; and on the onset of recrudescence, a second time treatment was given. We observed that recrudescent parasites in the combination treated mice failed to induce ECM at any stage after the first time treatment, thereby minimizing the risk of pathological damage to the brain. We demonstrate that treatment with our combination therapy for 4 days after the onset of ECM, reduced the percentage of CD3+CD4+Tbet+T cells (Th1) and increased the percentage of CD3+CD4+GATA3+ T cells (Th2) and CD4+CD25+Foxp3+ T cells (Treg) in the spleen on day 10 p.i in comparison to infected-untreated mice. CD4+Tbet+T cells have been shown to be necessary for the development of ECM and higher levels of CD4+CD25+Foxp3+ T cells have been associated with the prevention of the pathology (50–53). We also observed decrease in the expression of pro-inflammatory markers like IFN gamma, MIP 1beta, TNF alpha, IL 1beta, ICAM 1, CXCL 10, CXCR3 and CXCL 9 in the brain of combination treated mice in comparison to infected-untreated mice, which are known to have important roles in ECM development (54). Similarly, the serological levels of pro-inflammatory cytokines IFN gamma, TNF alpha, IL 12, IL 6 were lower in the combination treated mice whereas the levels of anti-inflammatory cytokines IL 4 and IL 10 were higher. Both IL 4 and IL 10 have been associated with protection from ECM development in *P. berghei* ANKA infected C57BL/6 mice (55, 56; 57). Thus, regulation of the immune response after combination treatment led to protection from ECM development in treated mice. Administering the same combination treatment regimen for a second time after recrudescence led to complete control of pathology and parasitemia in infected mice, and the splenic architecture returned to normal by day 90 p.i after which the observation was discontinued (**Figure S1**, **Tables S1, S2**). The present study has focused on improving the survival of *P. berghei* ANKA infected C57BL/6 mice, but we have limited understanding of how this is achieved by using the combination therapy. Therefore further studies are required to understand the mechanism of action in detail. The control of malaria will have to continue to rely on chemotherapy in the foreseeable future even if effective anti-malarial vaccines are developed (58). While the search for new anti-malarial therapeutics directed towards the identification of new drug targets in the parasite continues, efforts should also be directed towards developing therapies that can take into consideration the host/parasite relationship and many aspects of the disease and its complications. Given the significant involvement of the immune response due to immunopathological nature of the disease, an immunomodulation based ACT therapy involving careful selection of molecules that can work as an effective combination holds promise towards tipping the balance towards effective recovery in malaria.

## Data Availability Statement

The raw data supporting the conclusions of this article will be made available by the authors, without undue reservation.

## Ethics Statement

The animal study was reviewed and approved by Approval No. (IEAC/2017/B-287) by the Institute’s Animal Care and Use Committee (IACUC), National Centre for Cell Science, Pune and Committee for the Purpose of Control and Supervision of Experimental Animals (CPCSEA) India.

## Author Contributions

SK contributed to the conception and design of the study & obtained the grant. SM and GR performed the experiments and wrote the first draft of the manuscript. SM, SK, and BS edited and wrote the final manuscript. All authors contributed to manuscript revision, read, and approved the submitted version.

## Funding

SM was supported by UGC, Govt. of India and GR was supported by the grant to SK (BT/PR12943/MED/29/934/2015) from the Department of Biotechnology, Ministry of Science and Technology, Government of India.

## Conflict of Interest

The authors declare that the research was conducted in the absence of any commercial or financial relationships that could be construed as a potential conflict of interest.

## Publisher’s Note

All claims expressed in this article are solely those of the authors and do not necessarily represent those of their affiliated organizations, or those of the publisher, the editors and the reviewers. Any product that may be evaluated in this article, or claim that may be made by its manufacturer, is not guaranteed or endorsed by the publisher.
