# Supplementary material for: Oral Therapy Using a Combination of Nanotized Antimalarials and Immunomodulatory Molecules Reduces Inflammation and Prevents Parasite Induced Pathology in the Brain and Spleen of P. berghei ANKA Infected C57BL/6 Mice
Source: Front Immunol. 2022 Jan 13;12:819469. doi: 10.3389/fimmu.2021.819469 (PMC8793777; doi:10.3389/fimmu.2021.819469)
Supplement: Supplementary file 1 [file DataSheet_1.docx]

Supplementary Material

**SUPPLEMENTARY TABLES AND FIGURES.**

**Figures.**

**Figure S1.** Experimental mice were randomly divided into 2 different groups (n=12) after infection with 1X10^6^ *P. berghei* ANKA. The first group (infected-untreated) was treated orally for 4 days with 200 μL PBS per mice from day 6 to day 9 p.i. In the second group, treatment with a combination of ART+AC9+AND+CUR was first given from day 6 to 9 p.i, and again from day 24 to 27 p.i. after detection of recrudescence. A group of healthy uninfected mice were kept as a separate control group (uninfected). Histology of spleen sections from uninfected, infected-untreated and infected-combination treated group on day 10 and day 90 p.i. Data is representative of 3 independent experiments. Scale bar: 50μm.

**Tables.**

| Groups | Length (in cm) | Width (in cm) | Weight of spleen (in g) | Weight of mice (in g) | Splenic index |
| --- | --- | --- | --- | --- | --- |
| Uninfected | 0.833±0.058 | 0.233±0.058 | 0.220±0.010 | 19.833±0.850 | 0.011±0.001 |
| Infected-untreated (day 10 p.i) | 3.167±0.153*** | 0.653±0.021*** | 0.457±0.021*** | 16.863±0.451** | 0.027±0.001*** |
| Infected- combination treated (day 10 p.i) | 2.033±0.208*** | 0.427±0.015*** | 0.323±0.015*** | 20.133±0.473 ^n.s^ | 0.016±0.001*** |
| Infected-combination treated (day 90 p.i) | 1.000±0.100 ^n.s^ | 0.267±0.058 ^n.s^ | 0.263±0.015* | 22.400±0.954** | 0.012±0.001 ^n.s^ |

**Table S1.** Experimental mice were randomly divided into 2 different groups (n=12) after infection with 1X10^6^ *P. berghei* ANKA. The first group (infected-untreated) was treated orally for 4 days with 200 μL PBS per mice from day 6 to day 9 p.i. In the second group, treatment with a combination of ART+AC9+AND+CUR was first given from day 6 to 9 p.i, and again from day 24 to 27 p.i. after detection of recrudescence. A group of healthy uninfected mice were kept as a separate control group (uninfected). Spleen was collected from mice and the length and width was measured using a ruler on millimeter scale to assess splenomagaly. The weight of spleen was weighed using analytical scale and the splenic index was calculated as: Splenic index = (weight of spleen)/body weight of mice.Data is expressed as mean ±SD from 3 mice per group. [**P*<0.05, ***P*<0.01, ****P*<0.001, n.s: not significant].

| Groups | Sinusoidal congestion | Hemorrhages | Red pulp | Pigment laden macrophages | White Pulp | Parasitized RBCs |
| --- | --- | --- | --- | --- | --- | --- |
| Uninfected | Absent | Absent | Unremarkable | Absent | Unremarkable | Absent |
| Infected-untreated (day 10 p.i) | 1+ | 2+ | Widened | 2+ to 3+ | Widened | 2+ to 3+ |
| Infected- combination treated (day 10 p.i) | 1+, occasional | Focal, rare | Widened | 1+ to 2+ | Widened, prominent | 1+ to 2+ |
| Infected-combination treated (day 90 p.i) | Absent | Absent | Unremarkable | Absent | Unremarkable | Absent |

**Table S2.** Experimental mice were randomly divided into 2 different groups (n=12) after infection with 1X10^6^ *P. berghei* ANKA. The first group (infected-untreated) was treated orally for 4 days with 200 μL PBS per mice from day 6 to day 9 p.i. In the second group, treatment with a combination of ART+AC9+AND+CUR was first given from day 6 to 9 p.i, and again from day 24 to 27 p.i. after detection of recrudescence. A group of healthy uninfected mice were kept as a separate control group (uninfected). The histological findings between the groups in comparison to uninfected (control) group in the spleen of mice (3 mice per group) were tabulated. + is used to denote the severity and extent of the parameters at the site according to the following scale:- 1+ means < 25 % RBCs are parasitized, 2+ means 25-50 % RBCs are parasitized, 3+ means 50-75 % RBCs are parasitized, 4+ means > 75 % RBCs are parasitized, Absent - 0% RBCs parasitized.
